# Sharing reflections and expressing appreciation upon completing a decade as co-editor of the IJHPR

**DOI:** 10.1186/s13584-021-00497-3

**Published:** 2021-12-15

**Authors:** Bruce Rosen

**Affiliations:** Myers-JDC-Brookdale Institute, JDC Hill, Jerusalem, Israel

## Abstract

The Israel Journal of Health Policy Research (IJHPR) was launched in January 2012. In December 2021 it will be completing 10 years of continuous publication. I have had the privilege of serving as the journal’s co-editor in chief during this period, and after ten years of service I am now preparing to step down from that role. IJHPR achievements of which I am particularly proud include remaining true to its mission, attracting manuscripts from virtually all the Israeli institutions engaged in health policy research as well as many leading institutions abroad, widening the circle of Israeli professionals who are submitting manuscripts to journals, and helping many established Israeli academics expand their repertoires to include articles with strong policy components. Several people and organizations have helped make editing the IJHPR such a wonderful experience for me. They include IJHPR co-editor Avi Israeli, IJHPR associate editor Steve Schoenbaum, the Israel National Institute for Health Policy Research (which sponsors the journal), BioMed Central (which publishes the journal), the Myers-JDC-Brookdale Institute (my employer), my family (and particularly my wife, Laura Rosen), and the thousands of authors who have chosen to publish with the IJHPR. May the journal’s second decade be even better than its first one!

## Introduction

The Israel Journal of Health Policy Research was launched in January 2012. In December 2021 it will be completing 10 years of continuous publication. I have had the privilege of serving as the journal’s co-editor in chief during this period, and after ten years of service I am now preparing to step down from that role. I am using this editorial to share several reflections on the on the journal’s evolution and achievements. I will also use the editorial to thank several of the people who have helped make editing the IJHPR such a wonderful experience for me.

## Reflections

The journal’s mission, from its initiation until today, has always been “to promote intensive intellectual interactions among scholars and practitioners from Israel and other countries regarding all aspects of health policy… The ultimate aim of these intellectual interactions is to contribute to the development of health policy in Israel and around the world” [1]. A decade on, I am deeply gratified that we have remained true to that mission, and that, to a substantial degree, we have turned it into a reality.

I am particularly proud that over time we have succeeded in attracting high-quality manuscripts from a very high percentage of the Israeli researchers, universities, and research centers and others active in the health policy field. Moreover, I believe that by establishing a journal focused on Israeli health policy, and by being supportive of our authors, we have been able to widen the circle of Israeli professionals who are submitting manuscripts to journals. Many of these new authors hold analytic and managerial positions in government agencies and some of their articles have been among the journal’s most innovative and influential. No less importantly, the journal has helped many established and well-published Israeli academics expand their repertoires to include articles with strong policy components.

I am also proud that we have been extremely successful in recruiting scores of leading scholars from outside of Israel to write commentaries about the original research articles written by our Israeli authors [2]. These scholars, many of them from world-class institutions, have contributed greatly to the realization of one of the journal’s core aims: helping Israel learn from other countries and helping other countries learn from Israel.

Some of the key landmarks on the way have included getting the approval of the National Institute to develop concrete plans for the journal (2010), contracting with BioMed Central to serve as our publisher (2011), publishing our first set of articles (2012), being accepted into the Web of Science after just one year of publication (2013), launching our first article collection (2013), the 2015 publication of our first article by a Nobel Prize laureates [3], the publication in 2016 of our first articles by authors from both India [4] and China [5] (both of which are among our most highly cited articles to date), achieving the status of a Q2 journal (2017), and the 2019 publication of our first conference proceedings [6].

Other sources of gratification are not tied to particular dates. One of the most significant of these has been the opportunity to identify numerous submissions which constituted “diamonds in the rough” and then working closely with their authors to develop them into clear, insightful, and often important, articles. Another major source of gratification is that more and more leading Israeli researchers have become regular contributors to the journal—apparently due a mix of a satisfactory author experience and recognition of the journal’s growing stature and influence.

I’ve also greatly enjoyed working with my fellow editors on soliciting, editing, and publishing clusters of articles on a common theme. These have included the cluster of articles that the IJHPR published in 2018 about the relationship between research and policymaking [7–9] and the 2021 cluster on Israel’s rollout of COVID-19 vaccines [10–16].

Along with these achievements, it is important for me to also note two of the areas in which we have not yet done as well as I had expected. I do so, in part, because I hope that that they will get a boost when I step down from the co-EIC role.

The first area in which we could do better is that, despite the recruitment of several very fine assistant editors, we have not yet done enough to expand the journal’s core editorial team. I know that Avi Israeli, who is continuing on as editor-in-chief, has some concrete ideas on how to do so, and I expect that he will succeed in this, as he has in so many of his leadership roles over the years.

Another area in which we could be doing more is in connecting research and policy and strengthening ties between scholars in Israel and abroad. To that end, I have proposed to Avi, the Israel National Institute for Health Policy Research, and BioMed Central that I will transition to a new role as the IJHPR’s special projects editor. I am delighted that they have graciously agreed. In that new role I hope to launch several initiatives to strengthen the IJHPR’s policy and international linkages, and I hope that many of you will have opportunities to take part in them. Details to follow in the months ahead, so please stay tuned in.

## Expressions of appreciation

Over the past decade, co-editing the IJHPR has been one of the major foci of my life. Doing so has been consistently gratifying and at times exhilarating. I am happy to have this opportunity to acknowledge several of the key people who have contributed to my overwhelmingly positive experience.

I begin with a huge thank you to Avi Israeli, with whom I have shared the editor-in-chief role from the start. Avi has brought to the journal a uniquely broad understanding of health care in Israel and beyond, along with an unwavering commitment to scholarly excellence and policy relevance. It was Avi who proposed one of the journal’s signature features: the recruitment of leading international scholars to comment on the original research articles, typically written by Israeli authors. I also want to thank Avi for going along with many of the initiatives that I proposed, despite having questions or reservations about some of them.

Mid-way through the decade, Avi and I recruited Steve Schoenbaum to serve as our associate editor. This has proven to be one of our best decisions. Steve has played a central role in recruiting commentators and reviewers from beyond Israel. He has also worked intensively with many of our authors to improve their manuscripts—not just to the point where they could be accepted for publication, but also to the point where they would be widely read and cited.

A big thank you also goes to the Israel National Institute for Health Policy Research, the journal’s sponsor. The key players there have included the late Haim Doron, Shlomo Mor-Yosef, Orli Manor, Alik Aviram, Haim Bitterman, Ziva Litvak, and Gili Shilat. In addition to covering the costs of publication, the National Institute has provided the journal with a home base which is respected by all of Israel’s health care and academic institutions and played a major role in promoting the journal and disseminating its articles.

Thanks also to my employer, the Myers-JDC-Brookdale Institute for supporting me in my work on the journal. Both MJB executive directors under whom I have served—Jack Habib and Michael Hartal—recognized the importance of the IJHPR to me and to Israeli health care and have been supportive throughout.

My investment in the journal would not have been possible without the wonderful support of my family, and in particular the support of my life partner—Professor Laura Rosen. Laura has been a great cheerleader for the journal’s accomplishments and an invaluable adviser and support to me in facing the challenges, stresses, and difficult periods involved in being a journal co-editor.

Thanks also go to our publisher, BioMed Central, and particularly to Liz Hoffman, our journal development editor. Liz has been a terrific advocate for the journal, helping BMC understand, appreciate, and accommodate the IJHPR’s unique mission and needs.

Finally, I want to thank the thousands of authors who have submitted manuscripts to the Israel Journal of Health Policy Research over the past decade. A very special thank you goes to those who submitted articles in our first few years, before it was clear whether the journal would make it. I am also appreciative of those who chose to wait it out during those early years, and then became consistent and valued contributors to the journal, thereby playing major roles in its continued growth and success.

May the journal’s second decade be even better than its first one!

## Data Availability

Not applicable.

## Abbreviations

IJHPR: Israel Journal of Health Policy Research
BMC: BioMed Central
MJB: Myers-JDC-Brookdale Institute
